# Long term CMR follow up of patients with right ventricular abnormality and clinically suspected arrhythmogenic right ventricular cardiomyopathy (ARVC)

**DOI:** 10.1186/s12968-019-0581-0

**Published:** 2019-12-12

**Authors:** Giuseppe Femia, Christopher Semsarian, Mark McGuire, Raymond W. Sy, Rajesh Puranik

**Affiliations:** 1 Department of Cardiology, Royal Prince Alfred Hospital, Sydney, NSW Australia; 2 Agnes Ginges Centre for Molecular Cardiology Centenary Institute, The University of Sydney, Sydney, Australia; 3 Sydney Medical School Faculty of Medicine and Health, The University of Sydney, Sydney, NSW Australia

**Keywords:** Arrhythmogenic right ventricular cardiomyopathy (ARVC), Congenital heart disease, Task force criteria (TFC), Sudden cardiac death (SCD), Cardiovascular magnetic resonance (CMR)

## Abstract

**Background:**

The Task Force Criteria (TFC) for arrhythmogenic right ventricular cardiomyopathy (ARVC) was updated in 2010 to improve specificity. There was concern however that the revised cardiovascular magnetic resonance (CMR) criteria was too restrictive and not sensitive enough to detect early forms of the condition. We previously described patients with clinically suspected ARVC who satisfied criteria from non-imaging TFC categories and fulfilled parameters from the original but not the revised CMR criteria; as a result, these patients were not confirmed as definite ARVC but may represent an early phenotype.

**Methods:**

Patients scanned between 2008 and 2015 who had either right ventricular (RV) dilatation or regional dyskinesia satisfying at least minor imaging parameters from the original criteria and without contra-indication underwent serial CMR scanning using a 1.5 T scanner. The aims were to assess the risk of progressive RV abnormalities, evaluate the accuracy of the revised CMR criteria and the need for guideline directed CMR surveillance in at-risk individuals.

**Results:**

Overall, 48 patients were re-scanned; 24 had a first-degree relative diagnosed with ARVC using the revised TFC or a first-degree relative with premature sudden death from suspected ARVC and 24 patients had either left bundle branch morphology ventricular tachycardia or > 500 ventricular extra-systoles in 24-h. Mean follow up was 69+/− 25 months. The indexed RV end-diastolic, end-systolic volumes and ejection fraction were calculated for both scans. There was significant reduction in RV volumes and improvement in RV ejection fraction (EF) irrespective of changes to body surface area; − 11.7+/− 15.2 mls/m^2^, − 6.4+/− 10.5 mls/m^2^ and + 3.3 +/− 7.9% (*p* = 0.01, 0.01 and 0.04). Applying the RV parameters to the revised CMR criteria, two patients from the family history group (one with confirmed ARVC and one with a premature death) had progressive RV abnormalities satisfying major criteria. The remaining patients (*n* = 46) did not satisfy the criteria and either had normal RV parameters with regression of structural abnormalities (27,56.3%) or stable abnormalities (19,43.7%).

**Conclusion:**

The revised CMR criteria represents a robust tool in the evaluation of patients with clinical suspicion of ARVC, especially for those with ventricular arrhythmias without a family history for ARVC. For patients with RV abnormalities that do not fulfill the revised criteria but have a family history of ARVC or an ARVC associated gene mutation, a surveillance CMR scan should be considered as part of the clinical follow up protocol.

## Introduction

Arrhythmogenic right ventricular cardiomyopathy (ARVC) is an inherited cardiomyopathy with a variable and progressive phenotype associated with ventricular arrhythmias and/or sudden cardiac death (SCD) [1]. The clinical diagnosis is complex and dependent on several diagnostic categories including right ventricular (RV) imaging. The original Task Force Criteria (TFC) was created in 1994 and based on symptomatic index cases at the severe end of the disease spectrum and subjects with SCD [2]. Although the imaging category was believed to be specific, many thought it was not sensitive enough to detect early forms of the disease [3, 4].

Following these concerns, the TFC was revised in 2010 with significant modification to the imaging category through the creation of separate cardiovascular magnetic resonance (CMR) and transthoracic echo (TTE) criteria based on objective volumetric parameters [5]. It was hoped that the revised parameters would improve sensitivity without affecting specificity. However, we have previously shown a major reduction in the number of individuals satisfying the new CMR criteria suggesting that the changes may have made the revised criteria much more restrictive than the original version [6]. As a result, we wanted to evaluate a group of patients who satisfied non-imaging ARVC criteria and the original imaging criteria but not the revised CMR version [7]. These individuals had either a first-degree relative diagnosed with ARVC using the revised TFC, a first-degree relative (younger than 35 years of age) with premature sudden death due to suspected ARVC, non-sustained ventricular tachycardia (NSVT) with left bundle morphology or more than 500 ventricular extra-systoles in a 24-h period. Given that these patients harbour high risk clinical features for ARVC we hypothesize that they represent an early form of the condition and as such, require long term surveillance. Unfortunately, the revised TFC does not provide guidance for long term surveillance strategies, leaving the decision to the discretion of the clinician.

Therefore, we rescanned these patients using CMR imaging several years after the index CMR scan to evaluate the possibility of progressive RV abnormalities ie to assess whether these patients represent an early phenotype of ARVC, appraise the accuracy of the revised CMR criteria and provide recommendations for long term surveillance strategies.

## Methods

### Study population

We have previously described 61 patients with clinical features of ARVC who underwent CMR evaluation between January 2008 and December 2015 who satisfied major or minor criteria from non-imaging categories of the revised TFC and had CMR abnormalities that only satisfied the original imaging criteria but not the revised CMR version [6]. Hence, it was hypothesized that these patients may represent an “early” form of ARVC and as such were recommended to have ongoing long-term clinical and imaging follow up. Ethics approval was obtained from the Royal Prince Alfred Hospital Human Research Ethics Committee, Sydney, and all participants gave informed consent; Protocol number X17–0225 & HREC/17/RPAH/339.

**Fig. 1:**
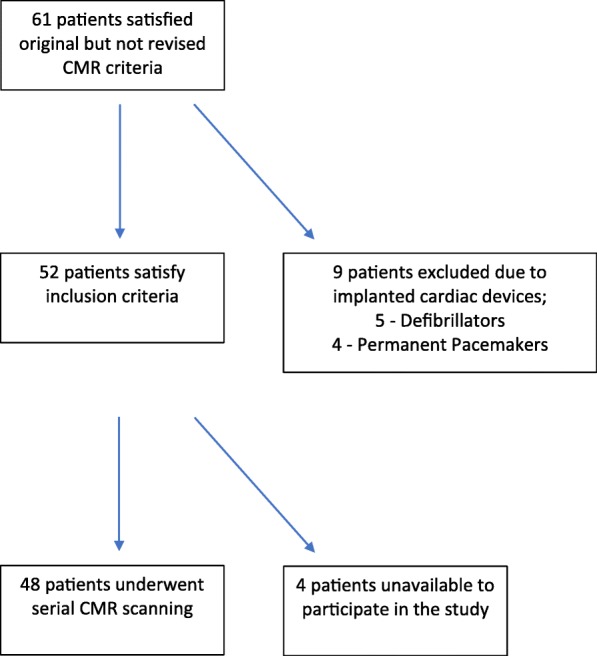
Flow Diagram

We obtained clinical data for all patients and performed serial CMR imaging provided there was no contra-indication such as the presence of an implanted cardiac device. In total, 48 (78.7%) patients were re-scanned; nine (14.8%) patients were excluded from CMR follow up due to an implanted cardiac device (five (8.2%) defibrillators and four (6.6%) permanent pacemakers) and four patients were unavailable to participate in the study; Fig. 1*.* For these patients, all available clinical data including echocardiograms, device checks, information on family history of ARVC or SCD and genetic testing were collected from the referring cardiologist or directly from the patients. Patient characteristics including age, height, weight and body surface area (BSA) were obtained. For the serial scans, we calculated the indexed end-diastolic and end-systolic volumes (EDVI and ESVI) and ejection fraction (EF) for the left ventricle (LV) and RV and examined the RV for regional wall motion abnormalities. For all scans, we report results using both the original imaging criteria and the revised 2010 CMR criteria; Table 1a*.*

**Table 1:** Abbreviated Original and Revised Task Force Criteria for the diagnosis of Arrhythmogenic Right Ventricular Cardiomyopathy (ARVC). A Criteria I – Imaging; B Criteria V – Arrhythmias; C Criteria VI – Family History. Revised from Marcus et al.

Original Task Force Criteria	Revised Task Force Criteria
A	
*I. Global or regional dysfunction and structural alterations; Imaging*	
Major	Major
-Severe dilatation and reduction of RV ejection fraction with no LV impairment -Localized RV aneurysms -Severe segmental dilatation of the RV	By 2D echo: Regional RV akinesia, dyskinesia or aneurysm and 1 of the following (end diastole and corrected for body size (Parasternal long axis /BSA)): - Parasternal long axis RVOT > 32 mm Parasternal long axis /BSA > 19 mm/m2 - Parasternal short axis RVOT > 36 mm Parasternal short axis /BSA > 21 mm/m2 -Fractional area change < 33% By CMR: Regional RV akinesia or dyskinesia or dyssynchrounous RV contraction and 1 of the following: -Ratio of RV EDV to BSA > 110 ml/m2 (male) or > 100 ml/m2 (female) -RV ejection fraction < 40%
Minor	Minor
-Mild global RV dilatation and/or ejection fraction reduction with normal LV -Mild segmental dilatation of the RV -Regional RV hypokinesis	By 2D echo: Regional RV akinesia or dyskinesia and 1 of the following (end diastole and corrected for body size (Parasternal long axis /BSA)) -Parasternal long axis RVOT > 29 to < 32 mm Parasternal long axis /BSA > 16 to < 19 mm/m2 -Parasternal short axis RVOT > 32 to 36 mm Parasternal short axis /BSA > 18 to < 21 mm/m2 -RV Fractional area change > 33% to < 40% By MRI: Regional RV akinesia or dyskinesia or dyssynchrounous RV contraction and 1 of the following: -Ratio of RV EDV to BSA > 100 to < 110 ml/m2 (male) or > 90 to < 100 ml/m2 (female) -RV ejection fraction > 40% to < 45%
B	
*V. Arrhythmias*	
Major	Major
	-Non-sustained or sustained ventricular tachycardia of left bundle-branch morphology with superior axis (negative or indeterminate QRS in leads II, III, and aVF and positive in lead aVL)
Minor	Minor
-Left bundle-branch block-type ventricular tachycardia (sustained and non-sustained) -Frequent ventricular extra-systoles (1000 per 24 h)	-Non-sustained or sustained ventricular tachycardia of RV outflow configuration, left bundle-branch block morphology with inferior axis (positive QRS in leads II, III, and aVF and negative in lead aVL) or of unknown axis - > 500 ventricular extra-systoles per 24 h
C	
*VI. Family history*	
Major	Major
-Familial disease confirmed at necropsy or surgery	-ARVC confirmed in a first-degree relative who meets current Task Force criteria -ARVC confirmed pathologically at autopsy or surgery in a first-degree relative -Identification of a pathogenic mutation categorized as associated or probably associated with ARVC in the patient
Minor	Minor
-Family history of premature sudden death (35 years of age) due to suspected ARVC -Familial history (clinical diagnosis based on present criteria)	-History of ARVC in a first-degree relative in whom it is not possible or practical to determine whether the family member meets current Task Force criteria -Premature sudden death (35 years of age) due to suspected ARVC in a first-degree relative -ARVC confirmed pathologically or by current Task Force Criteria in second-degree relative

2D, two dimensional; ARVC, arrhythmogenic right ventricular cardiomyopathy; BSA, body surface area; EDV, end-diastolic volume; LV, left ventricle; RV, right ventricle; RVOT, right ventricular outflow tract

### CMR protocol

Index CMR studies included multiple long and short axis (contiguous slices) cines for the RV and LV, aortic and pulmonary artery (PA) flows. Index scans included contrast studies as part of our standard clinical protocol but serial scans were non-contrast in accordance with the revised TFC criteria and performed with a 1.5 T CMR scanner (Achieva, Philips Healthcare, Best, The Netherlands) using a similar protocol including multiple long and short axis (contiguous slices) cines for the RV and LV and PA flow.

#### Volumetric volume and function assessment Utilising cine CMR

Specific image scanning parameters for serial CMR were as follows; balanced steady-state free precession (bSSFP) cine CMR images were acquired over a single breath-hold using the following imaging parameters: repetition time (TR) < 4 ms; echo time (TE) 1.5 ms; flip angle 60; slice thickness 8–10 mm; matrix 192,256; field of view 300–380 mm; and temporal resolution 40 ms. The following cine views were acquired: RV long axis, LV long axis, 4-chamber, biventricular short axis (including 9–12 contiguous ventricular slices), axial RV stack (including 9–12 contiguous ventricular slices), RV outflow tract (RVOT).

#### CMR flow calculation

Phase-sensitive (velocity encoding gradient [Venc] set at 200 cm/sec and adjusted to avoid aliasing), gradient-echo sequences (TR < 5 ms; TE < 3 ms; flip angle, 15°; slice thickness, 7 mm; field of view = 300–380 mm matrix, 256 9240, temporal resolution = 30 ms) were used to acquire PA flow data during a breath hold. The main PA midpoint was used as standardised imaging planes. Retrospective electrocardiogram (ECG) gating was utilised to acquire through plane data. A semi-automatic vessel edge-detection algorithm (Phillips Extender MR Work- space R2.6.3.1) with manual operator correction was utilised to obtain arterial blood flow from phase contrast images.

### Image analysis

All CMR studies were analysed by 2 experienced readers (SCMR level II accredited (GF) and III accredited (RP)). We have a high-volume centre for RV analysis and have previously reported on our RV reproducibility [8]. CMR evaluation software (OsiriX 3.93, Pixmeo, Bemex, Switzerland) http://www.osirix-viewer.com was used for viewing and analysis [9]. The endocardial borders of short-axis cine images were manually traced at end-systole and end-diastole and subsequently divided by body surface area to give the indexed RVEDVI and RVESVI as well as RVEF.

### Statistical analysis

Continuous data are expressed as mean +/− SD. TFC were expressed as dichotomous data. Any differences between groups were assessed by the paired t-test and *P* value < 0.05 was considered significant results.

## Results

### Demographics

A total of 48 patients (27 females, 56.3%) with diagnostic characteristics from non-imaging TFC criteria and RV structural abnormalities that satisfied the original but not the revised CMR criteria were re-scanned. Of these patients, 29 (60.4%) satisfied major non-imaging TFC criteria and 19 (39.6%) satisfied minor non-imaging TFC criteria; 16 (33.3%) patients had a first-degree relative with confirmed ARVC, 8 (16.7%) had a first-degree relative with premature sudden death due to suspected ARVC, 4 (8.3%) had a pathogenic mutation linked to ARVC (PKP2), 13 (27.1%) had ventricular tachycardia with left bundle branch morphology and 11 (22.9%) had more than 500 ventricular extra-systoles in a 24-h period. Patients were re-scanned at a mean of 66.9 +/− 24.9 months with a mean age of 45.0 +/− 16.7 years, where patients with a family history were significantly younger compared to those with ventricular arrhythmias (40.6 +/− 17.9 years vs 52.1 +/− 11.6 years, *p* = 0.015); baseline patient characteristics, Table 2. No significant difference was observed in the mean BSA between the first and second scans; 1.94 +/− 2.89 vs. 1.97 +/− 1.91, *p* = 0.48.

**Table 2:** Baseline Patient Characteristics and Index CMR Characteristic

	First Degree Relative with ARVC	First Degree Relative with premature death	Ventricular Tachycardia	Ventricular Extra-systoles	*P* value Family History vs Arrhythmia Group
*n (%)*	16 (33.3%)	8 (16.7%)	13 (27.1%)	11 (22.9%)	
*Age, years*	41.1 +/− 18.9	40.3 +/− 19.3	49.3 +/−13.1	55.4 +/− 9.2	0.015
*Sex, Female, n (%)*	11 (68.8%)	4 (50%)	6 (46.1%)	6 (54.5%)	0.561
*Treatment, n (%)*					
*Beta Blockers*	0	0	2 (15.4%)	2 (18.2%)	0.109
*Calcium Channel Blockers*	0	0	1 (7.7%)	0	1.000
*Electrical Ablation, n (%)*	0	0	3 (23.1%)	0	0.234
*Pathogenic mutation associated with ARVC, n (%)*					
*-PKP2*	4 (25%)	0	0	0	0.109
*Major Criteria from Another Category, n (%)*	16 (100%)	0	13 (100%)	0	0.556
*Minor Criteria from Another Category, n (%)*	0	8 (100%)	0	11 (100%)	0.556
Index CMR					
*RV Dilatation, n (%)*	4 (25%)	4 (50%)	6 (46.2%)	4 (36.6%)	0.7661
*RV Dyskinesia, n (%)*	12 (75%)	4 (50%)	7 (53.8%)	7 (63.4%)	0.7661
*2010 Major Imaging, n (%)*	1 (6.3%)	2 (25%)	1 (7.7%)	2 (18.2%)	1.000
*2010 Minor Imaging, n (%)*	15 (93.7%)	6 (75%)	12 (92.3%)	9 (81.8%)	1.000

### CMR imaging results

When comparing the RV parameters of the index and serial CMR scans for the overall group, the following changes were observed; RVEDVI 89.5 +/− 17.3 vs 79.9 +/− 19.9 ml/m^2^, mean difference – 9.6 +/− 12.1, p = 0.01; RVESVI 39.1 +/− 10.8 vs. 33.2 +/− 13.2 ml/m^2^, mean difference − 5.9 +/− 9.7, p = 0.01; RVEF 56.9 +/− 6.2% vs 59.8 +/− 8.6%, mean difference + 2.8 +/− 7.9%, *p* = 0.04; Table 3*.* When comparing the LV parameters; LVEDVI 79.2 +/− 16.2 vs. 72.6 +/− 18.2 ml/m^2^, mean difference − 6.4 +/− 9.6, *p* = 0.067; LVESVI 29.3 +/− 9.5 vs 25.9 +/− 10.7 ml/m^2^, mean difference − 3.5 +/− 6.7, *p* = 0.091; LVEF 63.4 +/− 5.9% vs 65.8 +/− 8.2%, mean difference 2.4 +/− 6.9%, *p* = 0.098.

**Table 3:** Serial CMR Characteristics

	First Degree Relative with ARVC *N* = 16	First Degree Relative with premature death *N* = 8	Ventricular Tachycardia *N* = 13	Ventricular Extra-systoles *N* = 11	*P* value Family History vs Arrhythmia Group
Serial CMR					
*Change in RVEDVI, ml*	−4.4 +/− 7.2	−6.8 +/− 6.8	−12.6 +/− 17.1	−11.2 +/− 8.7	0.099
*Change in RVESVI, ml*	−3.4 +/− 7.9	−4.2+/− 3.9	−8.3 +/− 13.3	−7.2 +/− 5.2	0.268
*Change in RVEF, ml*	+ 1.3 +/− 7.2	+ 3.1+/− 5.7	− 4.5 +/− 11.2	−3.5 +/− 4.5	0.438
*2010 CMR Major Criteria, n (%)*	1 (6.3%)	1 (12.5%)	0	0	0.489
*2010 CMR Minor Criteria, n (%)*	0	0	0	0	N/A

EDVI, Indexed end-diastolic volume; EF, ejection fraction; ESVI, Indexed end-systolic volume

From the index scans, 18 (37.5%) patients had isolated RV dilatation *without* regional RV dyskinesia and 30 (62.5%) had isolated regional RV dyskinesia *without* RV dilatation. In addition, 2 (4.2%) patients had RV late gadolinium enhancement (LGE) in the RVOT consistent with scar. Applying the RV parameters from the index scans, all 48 patients satisfied the original imaging criteria (6 (12.5%) major and 42 (87.5%) minor) but none satisfied the revised CMR criteria. From the serial scans, 28 (58%) patients had regression of RV abnormalities, 18 (38%) patients maintained the same degree of abnormality (either isolated RV dilatation or regional dyskinesia) but 2 (4%) patients had progressive RV abnormalities. After applying the parameters to the revised CMR criteria, 46 (96%) patients did not satisfy the criteria for ARVC and were classified as normal but 2 (4%) patients satisfied the revised major criteria and hence concluded as having developed definite ARVC. Of note, 20 (41.7%) patients would continue to satisfy the original imaging criteria.

**Fig. 2:**
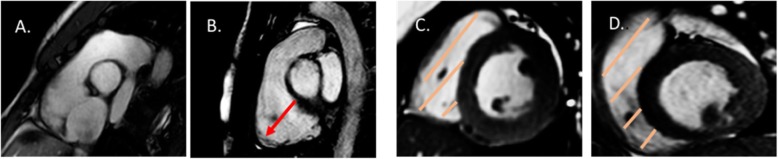
Two patients who developed progressive right ventricular (RV) abnormalities. Patient A satisfied major CMR criteria; indexed right ventricular end-diastolic volume (RVEDVI) > 110 ml/m^2^ and regional right ventricular dyskinesia (RV apex). Images A-B: Right ventricular outflow tract (RVOT) images – Index and serial scans. Red arrow shows dilated and dyskinetic right ventricular apex at end-systole. Patient B satisfied major CMR criteria; right ventricular ejection fraction (RVEF) < 40% and regional right ventricular dyskinesia (RVOT). Images C-D: Mid right ventricle short axis images – Index and serial scans. Orange strips show dilated right ventricle in end-systole

Amongst individuals referred with an ARVC family history (*N* = 24), 16 (66.7%) had a first-degree relative with confirmed ARVC using the revised criteria and 8 (33.3%) had a first-degree relative with a premature sudden death suspected of having ARVC. On the index scan, 8 (33.3%) patients had isolated RV dilatation and 16 (66.7%) had isolated regional RV dyskinesia; applying the RV parameters from the index scans, all 24 patients satisfied the original imaging criteria (3 (12.5%) satisfied the major criteria and 21 (87.5%)) satisfied the minor criteria but none satisfied the revised CMR criteria. The mean time to follow up was 64.6 +/− 21.7 months with a mean age of 40.8 +/− 18.6 years. Comparing the index and serial scans, the mean differences in RVEDVI, RVESVI and RVEF were − 7.8 +/− 10.4 mls/m^2^, − 5.5 +/− 9.2 mls/m^2^ and + 2.3 +/− 7.8% (*p* = 0.25, 0.28 and 0.29*.* From the serial scans, 12 patients were re-classified as normal, 10 maintained the same degree of abnormality (either isolated RV dilatation or regional RV dyskinesia) but 2 had progressive abnormalities with worsening RV dilatation or RV dyskinesia. Applying the RV parameters to the revised CMR criteria, 22 (91.7%) patients did not satisfy the revised CMR criteria and were classified as normal but 2 (8.3%) patients satisfied the 2010 major CMR criteria. Of the two patients who satisfied the 2010 major CMR criteria, one (patient A) had a first-degree relative (brother) with premature sudden death due to confirmed ARVC on post mortem histopathology and one (patient B) had a first-degree relative (mother) with premature sudden death due to suspected ARVC; Fig. 2*.* Of these 2 patients, neither had RV scar on the index scan or known pathogenic mutation associated with ARVC. Of note, of the 48 patients, 12 (50%) would continue to satisfy the original imaging criteria.

Amongst the individuals referred with NSVT or ventricular extrasystoles (*N* = 24), 13 (54.2%) had ventricular tachycardia with left bundle morphology and 11 (45.8%) had more than 500 ventricular extra-systoles in a 24-h period. On the index scan, 10 (41.7%) had isolated RV dilatation and 14 (58.3%) had isolated regional RV dyskinesia; applying the RV parameters all 24 satisfied the original imaging criteria (3 satisfied the major criteria and 21 satisfied the minor criteria) but none satisfied the revised CMR criteria. The mean time to follow up was 68.9 +/− 27.3 months with a mean age of 52.1 +/− 11.6 years. Comparing the index and serial scans, the mean differences in RVEDVI, RVESVI and RVEF were − 11.9 +/− 13.6 mls/m^2^, − 7.8 +/− 10.2 mls/m^2^ and + 4.1 +/− 8.6% (*p* = 0.03, 0.03 and 0.09)*.* From the serial scans, 16 patients were re-classified as normal and 8 maintained the same degree of abnormality (either isolated RV dilatation or regional dyskinesia); none of these patients satisfied the revised CMR criteria for ARVC but 8 (33.3%) patients would continue to satisfy the original imaging criteria.

### Clinical follow up

Amongst the patients scanned, only patients referred with ventricular arrhythmias were treated with antiarrhythmic therapy (20.8%; 5/24) at the time of follow up; four patients with a beta-blocker and one with a calcium channel blocker. In addition, three (12.5%) patients underwent successful electrical ablations for inducible RV ventricular tachycardia (RVVT). From the patients not scanned, nine patients were excluded due to an implanted cardiac device (five patients had an implanted defibrillator (secondary prevention) and four had a permanent pacemaker) and four were unavailable to participate in the study. Amongst this group, two patients were treated with antiarrhythmic therapy (beta-blockers) and two underwent successful RVVT ablation. On review of serial 2D TTE imaging and device checks, none of the patients had progressive RV abnormality satisfying the revised ARVC imaging criteria and none of these patients had documented shocks.

## Discussion

We found that despite satisfying the original imaging parameters and fulfilling other clinical features of ARVC, the risk of progressive RV abnormalities meeting the revised CMR criteria was low. In fact, there was evidence of RV remodeling with significant reductions in RV volumes irrespective of changes to body habitus, largely driven by patients with ventricular arrhythmias. Our results support previous studies and confirm the accuracy of the revised CMR criteria and the ability of CMR to appropriately risk stratify patients; however, the revised TFC does not provide guidance for long term follow up. We conclude that in patients who satisfy non-imaging criteria, such as those who are genotype positive-phenotype negative and who have any RV structural abnormalities, a standardized surveillance strategy that includes serial CMR imaging should be recommended.

For those with clinical suspicion of ARVC, confirming the diagnosis using the revised TFC is challenging particularly with recent concerns that the new objective imaging parameters have become too limiting and do not cater for early forms of the disease [4–6]. On the contrary however, our data demonstrates that reclassification of the index CMR by the revised criteria was accurate for 96% of patients, largely for patients with ventricular arrhythmias. Progression was noted in only two cases where patients had a family history of ARVC and potentially a genetic predisposition. Our results are in line with previous studies but extends the results with a larger cohort and the application of the revised CMR criteria [9]. In patients with isolated RVOT tachycardia, studies have shown instances where the RV structural abnormalities are reversible which is similar to the patients in our cohort. In fact, differentiating the two conditions can be difficult in early stages of ARVC particularly in patients with atypical phenotypes [10]. Regardless of the underlying condition, our results indicate that the risk of developing criteria fulfilling RV abnormalities for ARVC in patients with RV ventricular arrhythmias is low.

The risk of progressive RV abnormality was low in our cohort, particularly for patients without a family history of ARVC. Therefore, it is unlikely that these patients represent an early form of ARVC. For patients with a family history of ARVC however who are genotype positive but phenotype negative the long-term risk of disease progression remains uncertain and requires regular clinical review and serial CMR scanning.

## Study limitations

This is an observational prospective study that is limited by selection bias and relatively small sample size from a single centre. The modest sample size affects the reliability of our results and should be taken into account when interpreting them. In addition, due to the nature of the study, there was heterogeneity in follow up. As a consequence of our inclusion criteria, we may have missed RV abnormalities detectable by CMR in patients with implanted cardiac devices who were assessed and followed up without serial CMR scanning. Although noted, the study did not examine for the influence of medical therapy on RV remodeling and subsequently, we cannot make definitive conclusions on the underlying pathophysiology for RV remodeling over time. Finally, similar to other studies, we were limited by the lack of a single test or “gold standard” to definitely confirm the diagnosis of ARVC.

## Conclusions

In patients with clinical suspicion of ARVC and RV abnormalities that do not satisfy the revised CMR criteria, the risk of RV structural progression is low; particularly for those referred with ventricular arrhythmias. Our results support the accuracy of the revised CMR criteria as a diagnostic tool but the revised TFC are lacking in surveillance strategies for at risk patients. As such, for patients with a definite family history of ARVC and for those who are known to be genotype positive, we recommend comprehensive clinical follow up, including serial CMR scanning to evaluate RV parameters.

## Data Availability

The datasets used and/or analysed during the current study are available from the corresponding author on reasonable request.

## Abbreviations

ARVC: Arrhythmogenic Right Ventricular Cardiomyopathy
BSA: Body surface area
bSSFP: balanced steady state free precession
CMR: Cardiovascular magnetic resonance
ECG: Electrocardiogram
EDVI: End-diastolic volume index
EF: Ejection fraction
ESVI: End-systolic volume index
LGE: Late gadolinium enhancement
LV: Left ventricle/left ventricular
NSVT: Non-sustained ventricular tachycardia
RV: Right ventricle/right ventricular
RVOT: Right ventricular outflow tract
RVVT: Right ventricular ventricular tachycardia
SCD: Sudden cardiac death
TE: Echo time
TFC: Task Force Criteria
TR: Repetition time
TTE: Transthoracic echocardiogram
VENC: Velocity encoding
